# Phase 1, first-in-human study of TYRP1-TCB (RO7293583), a novel TYRP1-targeting CD3 T-cell engager, in metastatic melanoma: active drug monitoring to assess the impact of immune response on drug exposure

**DOI:** 10.3389/fonc.2024.1346502

**Published:** 2024-03-21

**Authors:** Anna Spreafico, Eva Muñoz Couselo, Anja Irmisch, Juliana Bessa, George Au-Yeung, Oliver Bechter, Inge Marie Svane, Miguel F. Sanmamed, Valentina Gambardella, Meredith McKean, Margaret Callahan, Reinhard Dummer, Christian Klein, Pablo Umaña, Nicole Justies, Florian Heil, Linda Fahrni, Eugenia Opolka-Hoffmann, Inja Waldhauer, Conrad Bleul, Roland F. Staack, Vaios Karanikas, Stephen Fowler

**Affiliations:** ^1^ Department of Medicine, Division of Medical Oncology and Hematology, Princess Margaret Cancer Centre, University Health Network and University of Toronto, Toronto, ON, Canada; ^2^ Department of Medical Oncology, Vall d’Hebron University Hospital and Vall d’Hebron Institute of Oncology (VHIO), Barcelona, Spain; ^3^ Roche Pharma Research & Early Development, Roche Innovation Center Zurich, Zurich, Switzerland; ^4^ Roche Pharma Research & Early Development, Roche Innovation Center Basel, Basel, Switzerland; ^5^ Department of Medical Oncology, Peter MacCallum Cancer Center and Sir Peter MacCallum Department of Oncology, The University of Melbourne, Melbourne, VIC, Australia; ^6^ Department of General Medical Oncology, Universitair Ziekenhuis (UZ), Leuven, Leuven, Belgium; ^7^ National Center for Cancer Immune Therapy and Department of Oncology, Copenhagen University Hospital, Herlev, Denmark; ^8^ Department of Medical Oncology, Clínica Universidad de Navarra and Immunology and Immunotherapy Program, Center for Applied Medical Research (CIMA), Pamplona, Spain; ^9^ Centro de Investigación Biomédica en Red Cáncer (CIBERONC), Instituto de Salud Carlos III, Madrid, Spain; ^10^ Department of Medical Oncology, Hospital Clínico Universitario de Valencia, INCLIVA Biomedical Research Institute, University of Valencia, Valencia, Spain; ^11^ Sarah Cannon Research Institute at Tennessee Oncology, Nashville, TN, United States; ^12^ Department of Medicine, Memorial Sloan Kettering Cancer Center and Weill Cornell Medical College, New York, NY, United States; ^13^ Department of Dermatology, University Hospital Zurich and University of Zurich, Zurich, Switzerland; ^14^ Roche Pharma Research & Early Development, Roche Innovation Center Munich, Penzberg, Germany

**Keywords:** TCB, bispecific, antibody, ADA, anti-drug antibody, immunogenicity, metastatic melanoma, TYRP1

## Abstract

**Introduction:**

Although checkpoint inhibitors (CPIs) have improved outcomes for patients with metastatic melanoma, those progressing on CPIs have limited therapeutic options. To address this unmet need and overcome CPI resistance mechanisms, novel immunotherapies, such as T-cell engaging agents, are being developed. The use of these agents has sometimes been limited by the immune response mounted against them in the form of anti-drug antibodies (ADAs), which is challenging to predict preclinically and can lead to neutralization of the drug and loss of efficacy.

**Methods:**

TYRP1-TCB (RO7293583; RG6232) is a T-cell engaging bispecific (TCB) antibody that targets tyrosinase-related protein 1 (TYRP1), which is expressed in many melanomas, thereby directing T cells to kill TYRP1-expressing tumor cells. Preclinical studies show TYRP1-TCB to have potent anti-tumor activity. This first-in-human (FIH) phase 1 dose-escalation study characterized the safety, tolerability, maximum tolerated dose/optimal biological dose, and pharmacokinetics (PK) of TYRP1-TCB in patients with metastatic melanoma (NCT04551352).

**Results:**

Twenty participants with cutaneous, uveal, or mucosal TYRP1-positive melanoma received TYRP1-TCB in escalating doses (0.045 to 0.4 mg). All participants experienced ≥1 treatment-related adverse event (TRAE); two participants experienced grade 3 TRAEs. The most common toxicities were grade 1–2 cytokine release syndrome (CRS) and rash. Fractionated dosing mitigated CRS and was associated with lower levels of interleukin-6 and tumor necrosis factor-alpha. Measurement of active drug (dual TYPR1- and CD3-binding) PK rapidly identified loss of active drug exposure in all participants treated with 0.4 mg in a flat dosing schedule for ≥3 cycles. Loss of exposure was associated with development of ADAs towards both the TYRP1 and CD3 domains. A total drug PK assay, measuring free and ADA-bound forms, demonstrated that TYRP1-TCB-ADA immune complexes were present in participant samples, but showed no drug activity *in vitro*.

**Discussion:**

This study provides important insights into how the use of active drug PK assays, coupled with mechanistic follow-up, can inform and enable ongoing benefit/risk assessment for individuals participating in FIH dose-escalation trials. Translational studies that lead to a better understanding of the underlying biology of cognate T- and B-cell interactions, ultimately resulting in ADA development to novel biotherapeutics, are needed.

## 1 Introduction

Treatment with novel immune checkpoint inhibitors has remarkably improved outcomes for patients with metastatic melanoma over the last decade (1). Notably, the use of anti-programmed cell death protein-1 (PD-1) alone or in combination with anti-cytotoxic T-lymphocyte-associated antigen-4 (CTLA-4) has resulted in 5-year overall survival rates ranging from 44–52% (2). More recently, the combination of anti-PD-1 with anti-LAG3 has shown superior clinical efficacy to anti-PD-1 alone (12-month progression-free survival: 47.7% vs 36.0%) (3). However, not all patients benefit from such therapeutic approaches, with many experiencing primary or acquired resistance to treatment (4, 5). Mechanisms of resistance to checkpoint inhibitor therapy, such as impaired interferon (IFN)-γ signaling and loss of antigen presentation, which lead to tumor evasion from the immune system, have been identified (6). T−cell engaging bispecific (TCB) molecules have the potential to bypass some of these evasion mechanisms, as they do not rely on pre-existing T-cell receptor recognition of tumor cells, but instead are able to activate any T-cell in the proximity of the tumor cell, independent of its specificity (7). While T-cell engagers are known to have transformative efficacy in hematological malignancies (8, 9), recent studies, such as those for tebentafusp in uveal melanoma and for tarlatamab in small cell lung cancer, have shown promise for T-cell engagers in solid tumors (10–12).

Tyrosinase-related protein 1 (TYRP1; gp75) is an enzyme functionally involved in melanin synthesis that is selectively expressed in the melanocyte lineage and the retinal pigment epithelium. TYRP1 is also a tumor antigen expressed in over 60% of cutaneous metastatic melanomas (internal analysis) and in 60–90% of uveal and sinonasal melanomas (13–15). The potential of TYRP1 as a therapeutic target has been explored previously in a phase 1 study evaluating the monoclonal antibody flanvotumab (IMC-20D7S), which showed that this treatment was well tolerated and had evidence of anti-tumor activity (16).

TYRP1-TCB is a novel TCB antibody comprising two TYRP1-binding domains and one CD3e-binding domain (2 + 1 format) (7, 17), enabling increased tumor antigen avidity and tumor cell killing. In cell culture and mouse tumor models, simultaneous binding to both targets leads to T−cell activation and concomitant T−cell mediated killing of TYRP1-positive melanoma cells (18).

Despite the clinical success of biotherapeutics, their use has sometimes been limited by the immune response mounted against them in the form of anti-drug antibodies (ADAs) (19). ADA onset can lead to serious consequences, such as neutralization of the drug and loss of efficacy, or adverse events (AEs) (20). Previous examples of ADA development have been reported when using tumor necrosis factor (TNF) inhibitors, such as infliximab and adalimumab (21). ADAs against other biologic therapeutics, such as those used to treat blood disorders (e.g., recombinant human erythropoietin) or cancers, have also been responsible for reduced efficacy and an increase in treatment discontinuation (22–24). In some instances, treatment discontinuation was directly related to an increase in ADA development and hence, reduced drug efficacy.

Rapid assessment of ADA incidence and its impact on drug exposure is important for understanding the duration of efficacy and ongoing patient benefit/risk assessment. Dual target binding competent assays measuring active TCB concentrations can rapidly highlight potential loss of exposure issues for clinical follow-up. Investigating the multifactorial nature of immunogenicity has led to several preclinical approaches currently being considered (25) and is of utmost importance for the development of improved immunotherapeutic modalities. Nevertheless, very few detailed studies have been published about the immunogenicity of T-cell engaging antibodies.

Based on preclinical data suggesting potent anti-tumor activity of TYRP1-TCB (18) and the prevalence of TYRP1 expression in melanoma, we conducted a first-in-human (FIH) phase 1 dose escalation study (BP42169; NCT04551352) to investigate the safety, tolerability, maximum tolerated dose (MTD)/optimal biological dose, and pharmacokinetics (PK) of TYRP1-TCB in patients with metastatic melanoma. Here, we present the results of this study and explore the immunogenicity of the molecule.

## 2 Methods

### 2.1 Study design and participants

This was a FIH, open-label, multicenter, phase 1 dose-escalation study with primary objectives of determining the safety, tolerability, and MTD/optimal biological dose of different dosing schemes of TYRP1-TCB in patients with metastatic melanoma. Characterization of PK, immunogenicity, and preliminary efficacy were secondary objectives. The study design comprised Part I, a single participant cohort (SPC) dose escalation, and Part II, a multiple participant cohort (*n ≥* 3) dose escalation in three dosing schemes (flat dosing, single step-up dosing [SUD], and fractionated dosing; **Supplementary Figure 1**). Additional participants could be added to any Part II cohort to further analyze the safety, PK, and pharmacodynamics (PD) of TYRP1-TCB. Eligible participants were aged ≥18 years, with unresectable metastatic TYRP1-positive melanomas, including uveal and mucosal melanomas, who had progressed on standard-of-care treatment or were intolerant to standard treatment. For inclusion, participants were required to have a central assessment of a freshly collected tumor biopsy for TYRP1 positivity. For the initial participants (SPCs; **Supplementary Figure 1**), a recent archival biopsy was acceptable. Key exclusion criteria included active, acute, or chronic inflammatory diseases of the skin affecting more than 5% of the body surface, e.g., psoriasis, atopic dermatitis, history of Stevens-Johnson syndrome, toxic epidermal necrolysis, or drug rash with eosinophilia and systemic symptoms. Other inflammatory diseases, including vitiligo, were allowed. Participants with medical conditions at risk of defects in the Bruch’s membrane of the eye (e.g., age-related macular degeneration) or a history of recurrent uveitis or medical conditions that are associated with frequent uveitis were excluded from study participation. All enrolled participants provided written informed consent. This study was approved by each center’s ethics committee or institutional review board and was conducted in conformance with the Declaration of Helsinki, International Conference on Harmonization Guidelines for Good Clinical Practice, and appropriate laws and regulations.

### 2.2 Study treatment

Participants received escalating doses of TYRP1-TCB (0.045 to 0.4 mg) intravenously (IV) on Day 1 of each 21-day cycle for up to 17 cycles (1 year). The FIH starting flat dose of 0.045 mg was derived from a MABEL approach based on the 30% pharmacological activity (PA) level in an *in vitro* activity assay, utilizing human peripheral blood mononuclear cells and a high TYRP1-expressing human melanoma cell line. A PA level of 30% has been reported as an acceptable approach for the starting dose selection of CD3 bispecific antibodies (26). Participants enrolled into the SPCs received 0.045, 0.135, and 0.4 mg TYRP1-TCB every 21 days (**Supplementary Figure 1**). Cohort A1 received 0.4 mg TYRP1-TCB every 21 days (flat dosing). Cohort B1 received 0.1 mg TYRP1-TCB in Cycle 1 followed by 0.4 mg in subsequent 21-day cycles (single SUD). Cohort C1 received TYRP1-TCB twice in Cycle 1 (Day 1, 0.05 mg; Day 8, 0.1 mg), followed by 0.4 mg 14 days later (Cycle 2, Day 1) and every 21 days thereafter (fractionated dosing). Frequent PK, ADA, and cytokine assessments were undertaken during the initial cycles of treatment. An overview of PK, ADA, and cytokine sample collection, and blood flow cytometry can be found in **Supplementary Table 1**.

### 2.3 Safety assessments

Characterization of the safety and tolerability of TYRP1-TCB was a primary objective of the study. AEs were graded according to National Cancer Institute Common Terminology Criteria for Adverse Events version 5.0 and cytokine release syndrome (CRS) according to the American Society for Transplantation and Cellular Therapy consensus grading (27). Preliminary treatment efficacy was a secondary endpoint and was assessed using Response Evaluation Criteria in Solid Tumors (RECIST) version 1.1.

### 2.4 TYRP1 expression

TYRP1 protein expression was centrally assessed at a College of America Pathologist-Clinical Laboratory Improvement Amendments certified laboratory (Ventana Medical Systems, Tucson, AZ, USA) with an investigational TYRP1 immunohistochemistry (IHC) assay, using the Abcam clone EPR13063 as primary antibody (Abcam, Cambridge, UK). Testing was performed on the Ventana Benchmark Ultra platform with the Optiview DAB kit as the detection system (Ventana Medical Systems, Tucson, AZ, USA). Two different cut-offs for TYRP1 tumor cell expression were applied to determine participant eligibility. For the first 12 enrolled participants, at least 25% TYRP1-tumor cell expression at intensities equal to or greater than IHC 1+ was applied. After protocol amendment, based on emerging data from the ongoing trial and additional validation efforts undertaken as part of the IHC development, the cut-off was lowered to at least 1% TYRP1-tumor cell expression at intensities equal to or greater than IHC 1+ for the remaining participants (*n* = 8). The H score was obtained by the formula: 3 × percentage of IHC 3+ (strongly stained cells) + 2 × percentage of IHC 2+ (moderately stained cells) + 1 × percentage of IHC 1+ (weakly stained cells), giving a range of 0 to 300.

### 2.5 Cytokine and other PD assessments

Plasma samples for cytokine analysis were collected at predose and at the end of infusion (EOI) on Days 2, 3, 8, and 15 in Cycle 1–3, as this was when the greatest drug-related changes in cytokines were expected. Assessments in subsequent cycles were increasingly sparse. A full list of the samples collected can be found in **Supplementary Table 1**. Samples were analyzed for cytokines and chemokines (i.e., interleukin [IL]-2, IL-6, IL-8, IL-10, IFN-γ, soluble IL-2R, CXCL10 and TNF-α) using validated multiplex immunoassays on a ProteinSimple Ella platform (Microcoat Biotechnologie GmbH, Bernried am Starnberger See, Germany). In addition, assessments of various immune cell populations in blood and intratumoral CD8 changes were evaluated in paired biopsies (data not shown). No receptor occupancy assessments were conducted.

### 2.6 PK assessments

Two bioanalytical ligand-binding assays were used for PK assessment of the concentration of target-binding competent drug and total drug, respectively.

The concentration of target-binding competent drug (defined as TYRP1-TCB molecules competent in binding both TYRP1 and CD3) was determined using a TYRP1-TCB serial sandwich enzyme-linked immunosorbent assay (ELISA) (28, 29). This active drug assay employed a biotinylated recombinant human TYRP1 capture protein immobilized on a streptavidin-coated microplate and a mouse digoxigenylated monoclonal antibody directed against the CD3 binding domain of TYRP1-TCB as the detection antibody, followed by the addition of a secondary detection solution, with horseradish peroxidase-conjugated sheep anti-digoxigenin Fab fragments (anti-Dig-POD) (**Supplementary Figure 2**). The signal was detected with the addition of a 3,3,5,5-tetramethylbenzidin (TMB) peroxidase substrate solution and the reaction was stopped with the addition of sulfuric acid. Color development was measured by reading at 450 nm for detection absorbance and 690 nm for reference absorbance. The measured absorbance signal was proportional to the TYRP1-TCB concentration in the human serum sample. The lower limit of quantification in human serum was 1.00 ng/mL for the target-binding competent drug. Key aspects for ensuring appropriate active/target binding competent drug quantification were considered (30–32), especially to avoid dissociation of the drug-target or drug-ADA complexes present in the sample.

In addition, an exploratory total drug PK (ELISA) assay was applied to selected samples (**Supplementary Figure 2**). Human serum samples were incubated with 0.1 M glycine hydrochloride pH 2.0 to dissociate ADAs, or any potential other binding partners, from TYRP1-TCB. Acidified samples were mixed with a biotinylated capture antibody directed against a PGLALA mutation in TYRP1-TCB (Fc part) and a digoxigenylated detection antibody directed against the CD3 binding site in TYRP1-TCB, neutralized with 0.5 M TRIS buffer pH 8.5 and incubated at room temperature. The immune complexes formed were transferred to a streptavidin-coated plate and incubated to immobilize the immune complexes via the biotin-labeled capture antibody. Unbound substances (e.g., reagents or matrix components) were removed by repeated washing. Immobilized immune complexes were incubated with anti-Dig-POD and visualized by addition of TMB as peroxidase substrate. The lower limit of quantification in human serum was 5.2 ng/mL for total drug. Only a subset of the serum PK samples were analyzed in this way: those from subjects who had been treated with 0.4 mg TYRP1-TCB every-3-weeks and were ADA positive.

### 2.7 Immunogenicity assessment: ADA assays

ADA testing was performed using a 3-tiered approach (validated screening, confirmation, and titer assay) detecting anti-TYRP1-TCB antibodies of all immunoglobulin classes in human serum.

For screening (tier 1), biotinylated and digoxigenylated TYRP1-TCB were used as capture and detection reagents, respectively, along with anti-Dig-POD and TMB as substrate. A 1:1 mixture of two monoclonal antibodies directed against both binding sites (anti-TYRP1 and anti-CD3) of TYRP1-TCB was used as a positive control. The assay sensitivity of the screening assay was 9.27 ng/mL for the positive control.

In human serum samples identified as potentially positive in the screening assay, the presence of specific anti-TYRP1-TCB antibodies was confirmed or excluded using the same ELISA method with an appropriate immuno-competition step (addition of excess TYRP1-TCB, confirmation assay, tier 2). Samples were confirmed as containing specific anti-TYRP1-TCB antibodies if there was a signal reduction greater than or equal to 36.1% in the presence of TYRP1-TCB. The antibody titer of a confirmed positive sample was determined by the analysis of a dilution series of the sample, using the screening assay. The titer was reported as the reciprocal of the greatest dilution (inclusive of the assay, the minimum required dilution of 100) that resulted in a response equal to or above the titer cut-off.

### 2.8 Characterization of ADA specificity: domain detection analysis

For characterization of the ADA specificity and to evaluate whether the immune response was directed against the anti-CD3 or anti-TYRP1 binding domains within TYRP1-TCB, a domain detection assay approach was applied (33).

Negative control (pooled human serum), positive controls/quality controls (1:1 mixture of two monoclonal antibodies directed against anti-TYRP1 and anti-CD3 of TYRP1-TCB), and study samples were incubated with 0.1 M glycine hydrochloride pH 2.0. Acidified samples were mixed with the capture antibody, either a biotinylated monoclonal antibody against the CD3 binding site (Fab fragment) for detection of ADAs against the CD3 domain or a biotinylated monoclonal antibody against the TYRP1 binding site (Fab fragment) for detection of ADAs against the TYRP1 domain, and detection antibody TYRP1-TCB-Dig. After incubation, samples were neutralized with 0.5 M TRIS buffer pH 8.5 and further incubated. Formed immune complexes were transferred to a streptavidin-coated 96-well plate and incubated for 1 hour (h) to immobilize the immune complexes via the biotin-labeled capture antibody. Immobilized immune complexes were incubated with anti-Dig-POD and visualized by the addition of TMB.

### 2.9 Jurkat NFAT reporter cell assay

Functional activity of TYRP1-TCB in human serum was tested in a Jurkat NFAT reporter cell assay with CHO-K1 TYRP1 cells. Jurkat NFAT reporter cells (Promega, Madison, WI, USA) were cultured in RPMI 1640 (Thermo Fisher Scientific, Waltham, MA, USA) containing 10% FBS, 2 g/L glucose (Merck KGaA, Darmstadt, Germany), 2 g/L NaHCO3 (Merck KGaA), 25 mM HEPES (Thermo Fisher Scientific), 1% GlutaMax (Thermo Fisher Scientific), 1 × NEAA (Merck KGaA), 1% SoPyr (Merck KGaA) (Jurkat NFAT medium) at 0.1–0.5 Mio cells/mL. CHO-K1 TYRP1 cells were cultured in DMEM + GlutaMAX (1×) (Thermo Fisher Scientific) containing 10% FBS and 6 µg/mL puromycin (Invivogen, San Diego, CA, USA). The assay was performed in advanced RPMI 1640. For the assay, the CHO-K1 TYRP1 cells were resuspended in assay medium and 30,000 viable cells were seeded per well in a white flat bottom 96-well plate. 60,000 viable Jurkat NFAT reporter cells were seeded per well, corresponding to an effector-to-target ratio of 2:1, then 140 µL of the TYRP1-TCB containing participant serum was added per well, corresponding to 70% of the total volume. Additionally, 2% end-volume of GloSensor cAMP Reagent (E1291, Promega) was added to each well. After 4 h incubation time, luminescence was measured using a Tecan (Männedorf, Switzerland) Spark10M device.

## 3 Results

### 3.1 Prevalence of TYRP1 expression

Overall, tumor samples from 207 potential participants were centrally prescreened for TYRP1 expression. In order to enrich for potential participants with a higher likelihood of a positive fresh sample and create a pool of potential future participants, archival biopsies were prescreened as a first step in most cases (*n* = 187).

Overall, 68% of prescreening samples expressed TYRP1 at any level (at least 1% TYRP1 tumor cell expression at staining intensities equal to or greater than IHC 1+) and 51% of participants expressed TYRP1 when using a 25% TYRP1 tumor cell expression cut-off at staining intensities equal to or greater than IHC 1+. Prevalence of TYRP1 expression differed between melanoma subtypes (**Supplementary Table 3**), with uveal melanoma showing the highest frequency of positive samples with any TYRP1 expression (91%, *n* = 41 of 45) followed by mucosal melanoma (74%, *n* = 20 of 27) and cutaneous melanoma (59%, *n* = 80 of 135). For the enrolled participants (*n* = 20), TYRP1 expression at baseline was as follows: 3 had a H-score <100, 4 had a H-score of 100–200, and 13 had a H-score >200.

### 3.2 Participant characteristics

Twenty participants with advanced TYRP1-positive melanoma (cutaneous, *n* = 10; uveal, *n* = 6; mucosal, *n* = 3; unknown primary, *n* = 1) were enrolled and received TYRP1-TCB (participant characteristics are presented in **Table 1**); 19 of these participants had received at least one prior systemic therapy that included an anti-PD-1 antibody. Three participants were enrolled into SPCs, 7 into Cohort A1 (flat dosing), 5 into Cohort B1 (single SUD), and 5 into Cohort C1 (fractionated dosing). One participant (Participant #1) was intra-participant dose-escalated, receiving 0.045 mg in Cycle 1 and Cycle 2 and 0.135 mg in Cycle 3 and Cycle 4. Participants received an average of six doses (range: 1–17 doses) of study treatment before discontinuation, with 1 participant receiving the full course of treatment (1 year; **Supplementary Figure 3**). The MTD and/or optimal biological dose were not reached.

**Table 1:** Study participant clinical characteristics.

*N* (%) unless stated		Total (*N* = 20)
Gender	Male	11 (55)
	Female	9 (45)
Mean age, years (range)		59.5 (38–76)
Race	White	18 (90)
	Asian	1 (5)
	American Indian or Alaskan native	1 (5)
Weight, mean kg (range)		80 (51–125)
ECOG performance status	0	13 (65)
	1	7 (35)
Melanoma type	Cutaneous	10 (50)
	Uveal	6 (30)
	Mucosal	3 (15)
	Unknown primary	1 (5)
Disease stage (AJCC)	IV M1a M1b M1c M1d M1	20 (100) 2 (10) 3 (15) 11 (55) 2 (10) 2 (10)
Baseline LDH	>ULN	13 (65)
	More than 2x ULN	5 (25)
Most common baseline condition	Nausea	8 (40)
	Hypothyroidism	6 (30)
	Hypertension	5 (25)
	Fatigue	5 (25)
Mean time since diagnosis, years (range)		2.6 (1.1–10.5)
Median number of prior systemic treatments, *n* (range)		2 (0–7)
Prior systemic therapy	Immunotherapy Anti-PD-1 + anti- CTLA-4 Anti-PD-1 Investigational Others	14 (70) 2 (10) 1 (5) 10 (50) 1 (5)
	Chemotherapy	4 (20)
	Targeted therapy	1 (5)
	None	1 (5)

ECOG, Eastern Cooperative Oncology Group; LDH, lactate dehydrogenase; ULN, upper limit of normal.

### 3.3 Safety and tolerability

All participants had at least one treatment-related AE (TRAE) of any grade (**Table 2**), with the most common TRAEs being CRS (12 participants), rash (11 participants), and fatigue (5 participants). No grade 4 or 5 AEs or TRAEs leading to discontinuation were reported. In total, 40% of participants experienced a serious AE, all of which were grade 1–2 CRS events that required extension of hospitalization for treatment and monitoring. Most participants (*n* = 19) discontinued from study treatment due to progressive disease, and 1 participant withdrew consent. One participant in Cohort B1 experienced a grade 3 dose-limiting toxicity with increased liver function tests after the 0.4 mg TYRP1-TCB dose. Only three grade 3 TRAEs were reported (two events of anemia in 1 participant from Cohort A1 and one event of increased liver function test in another participant).

**Table 2:** Treatment-related adverse events in ≥2 participants.

*N* (%) of participants	Part I	Part II			Total (*N* = 20)
	Single participant cohorts (*N* = 3)	Cohort A1 (*N* = 7)	Cohort B1 (*N* = 5)	Cohort C1 (*N* = 5)	
Any TRAE	3 (100%)	7 (100%)	5 (100%)	5 (100%)	20 (100%)
CRS* Grade 1 Grade 2	2 1 1	6 2 4	4 2 2	0 0 0	12 (60%)
Rash** Grade 1 Grade 2	2 2 0	3 2 1	3 1 2	3 2 3	11 (55%)
Fatigue	0	3	1	1	5 (25%)
Pruritis	0	1	2	1	4 (20%)
Pyrexia	0	1	2	1	4 (20%)
Decreased appetite	0	2	1	0	3 (15%)
Skin exfoliation	0	1	2	0	3 (15%)
Infusion-related reaction	2	1	0	0	3 (15%)
Hypophosphatemia	0	1	1	0	2 (10%)
Abdominal pain upper	2	0	0	0	2 (10%)
ALT increased	1	0	1	0	2 (10%)
Anaemia	0	1	0	1	2 (10%)
AST increased	1	0	1	0	2 (10%)
Asthenia	1	1	0	0	2 (10%)
Chills	0	0	1	1	2 (10%)
Dry skin	0	1	1	0	2 (10%)
Erythema	0	0	2	0	2 (10%)
Uveitis	0	1	1	0	2 (10%)
Any serious TRAE***	1 (33%)	5 (71%)	2 (40%)	0	8 (40%)

All TRAEs were grade 1 or grade 2 with the exception of two events of grade 3 anemia and one event of grade 3 elevated liver function test (not shown in this table). Grades are listed for the most common TRAEs of CRS and rash only.

*All CRS events were grade 1 or grade 2 per ASTCT criteria (20). Premedication with corticosteroids was not required per protocol. One case of CRS required tocilizumab (anti-IL-6 receptor) treatment.

**Rash is a composite term including the preferred terms rash, rash maculopapular, and rash pruritic.

***All serious TRAEs were grade 1 or 2 CRS events that required prolonged hospitalization.

ALT, alanine transaminase; AST, aspartate transaminase; ASTCT, American Society for Transplantation and Cellular Therapy; CRS, cytokine release syndrome, IL-6 receptor, interleukin-6; TRAE, treatment-related AE.

The most common TRAE was CRS, which has been described for other TCB molecules. CRS of grade 1 or 2 occurred in 60% of participants overall (*n* = 12) after the first and/or second dose, with 2/3 participants in the SPCs, 6/7 participants in Cohort A1, 4/5 participants in Cohort B1, and no participants in Cohort C1 experiencing CRS. Thus, the highest dose of 0.4 mg induced CRS in the majority of participants (7/8) when given as first dose, but not after starting two lower doses (0.05 and 0.1 mg; **Table 3**) in the fractionated dosing cohort. In contrast, the single SUD schedule of 0.1 mg in Cycle 1 followed by 0.4 mg 3 weeks later (Participant #11 to #15) was insufficient for avoidance of CRS. The most common symptoms associated with CRS were pyrexia, hypotension, and diarrhea. Most cases were managed with antipyretics, IV fluids, and steroids. Three participants in Cohort A1 required treatment with corticosteroids (≤100 mg methylprednisolone), one of whom required one dose of tocilizumab due to grade 2 CRS that was refractory to one dose of IV corticosteroids. After the occurrence of the first grade 2 CRS in Participant #3, CRS prophylaxis was implemented and subsequent participants (cohorts A1, B1, and C1) received corticosteroid-free pretreatment with IV fluids, anti-inflammatories (e.g., acetaminophen), and an antihistamine. No relationship between ADA titer and AEs such as CRS was observed (**Table 3**; * symbolize CRS occurrences). Rash, the second most common TRAE, was grade 1–2 and reported in 2/3 participants in the SPCs, in 3/7 participant in Cohort A1, and in 3/5 participants in cohorts B1 and C1 (**Table 2**).

**Table 3:** Overview of ADA appearance and CRS incidence by participant, cohort, and cycle number.

Participant number	Dose and schedule	Treatment cycle														
		1	2	3	4	5	6	7	8	9	10	11	12	13	14	15
1	0.045 mg (C1 + 2), 0.135 mg (C3 + 4) intra-participant dose escalated (Part I)			*	*											
2	0.135 mg flat dose (Part I)															
3	0.4 mg flat dose (Part I)	**	900	900	2700		2700		2700	2700	2700	2700	2700	2700	2700	8100
4	0.4 mg flat dose (Part II, cohort A1)	**			8100	2700	2700									
5																
6		*	900	300	300											
7		**		2700	2700	2700	2700	2700	2700	2700	900	2700	2700	2700		
8		**		100	8100	2700	2700	8100	8100	8100	8100	8100				
9		**	300	100	300	900	900	900	2700	2700	2700	2700	2700	900	900	
10		100 *														
11	0.1–0.4 mg single step-up dosing (Part II, cohort B1)	**	**													
12		*		*												
13		**	300													
14																
15		*	*													
16	0.05/0.1/0.4 mg fractionated dosing (Part II, cohort C1)															
17																
18																
19				100	2700	24300	72900	24300								
20																

* = CRS grade 1.

** = CRS grade 2.

Blue cells: participant on treatment and ADA negative; red cells: participant on treatment and ADA positive with ADA titer listed; orange cells: low-level pre-existing ADA signal.

Note that Participant #1 had an intra-participant dose-escalation from 0.045 mg to 0.135 mg in Cycle 3 and displayed grade 1 CRS in cycles 3 and 4.

ADA, anti-drug antibody; C, Cycle; CRS, cytokine release syndrome.

### 3.4 Clinical activity

No complete or partial responses per RECIST version 1.1 were reported in the 17 participants with a post baseline tumor assessment. The best overall response was stable disease (with no notable decreases in sum of target lesion diameter), which was observed in 12 of 17 participants. In all participants with stable disease, this was present at the first tumor scan 6 weeks after start of study treatment. Three participants stayed on study with stable disease for more than 30 weeks, with 1 participant completing the 1-year per-protocol treatment period (**Supplementary Figure 3**). At the time of study termination, 12 participants had died due to disease progression, 1 participant had experienced symptomatic deterioration, 1 participant had withdrawn consent, and 6 participants were alive.

### 3.5 Pharmacodynamic modulations upon treatment

**Figure 1:**
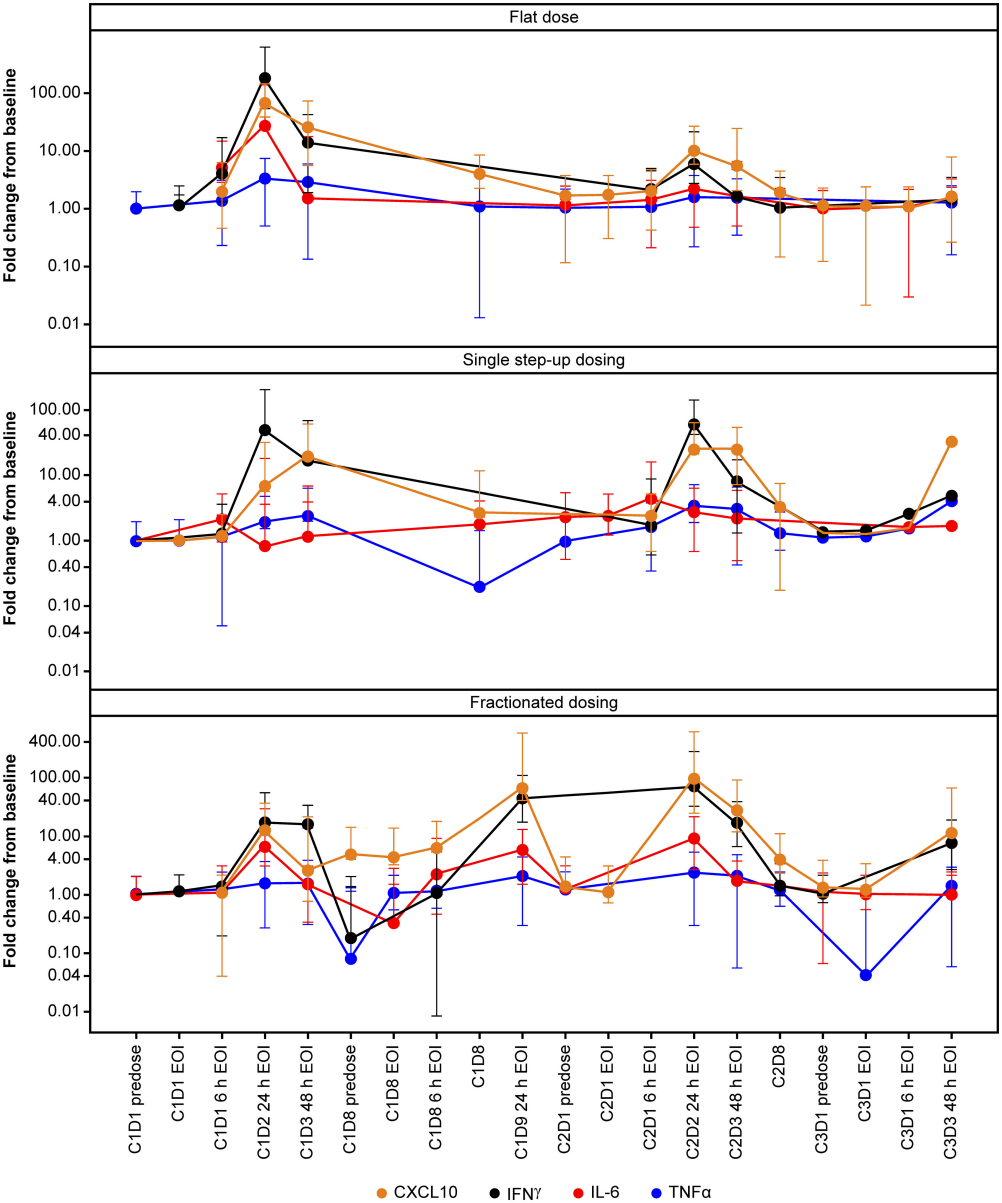
Temporal profile of cytokines. Profile of selected cytokines in the first three cycles of TYRP1-TCB treatment. IFN-γ and CXCL10 increases in all cohorts whereas IL-6 (and TNF-α) levels are maintained over time in the fractionated dosing cohort. C, cycle; CXCL10, C-X-C motif chemokine ligand 10; D, day; EOI, end of infusion; h, hours; IFN-γ, interferon-gamma; IL-6, interleukin-6; TCB, T-cell engaging bispecific; TNF-α, tumor necrosis factor-alpha; TYRP1, tyrosinase-related protein 1.

Cytokines, including IL-6, IFN-γ, TNF-α, and CXCL10 chemokine, were measured in plasma as a PD readout of TYRP1-TCB. **Figure 1** shows the levels and kinetics of measured cytokines per dosing regimen, i.e., flat, single SUD, and fractionated dosing. Upon first infusion, all cytokines were induced in a dose-dependent manner. Participants who received 0.4 mg flat dosing (*n* = 8) showed peak levels of cytokines 24 h post-first infusion, with detectable but reduced levels upon second infusion (Cycle 2). In contrast, participants treated with single SUD or fractionated dosing had detectable post-dose cytokine increases until the third or fourth infusion (Cycle 3). Importantly, participants treated with fractionated dosing had elevated levels for IFN-γ and CXCL10 until the 0.4 mg target dose was achieved at Cycle 2, whereas IL-6 and TNF-α levels seemed to be maintained over time.

**Figure 2:**
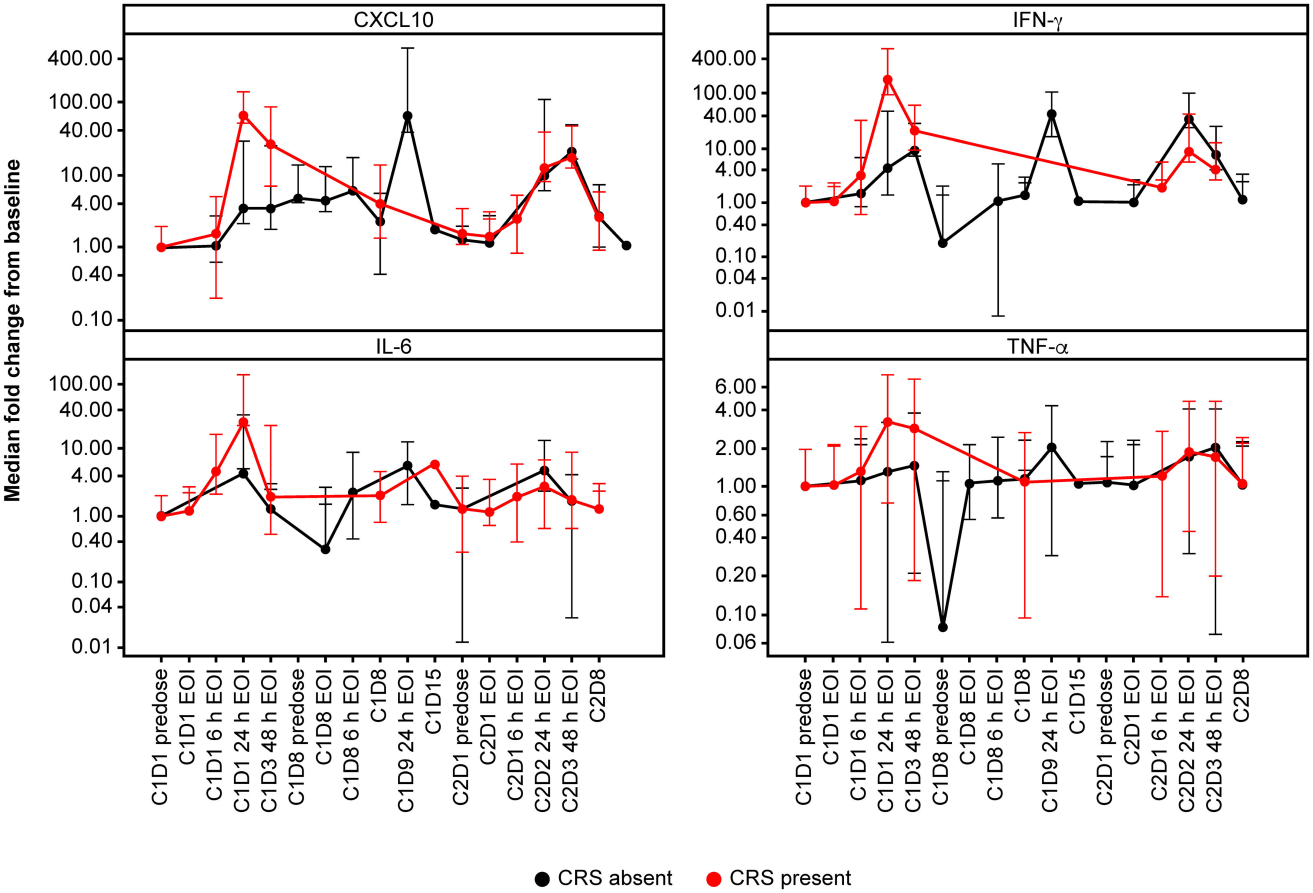
Cytokine profile according to CRS. Cytokine profiles according to presence (red) or absence (black) of CRS. Patients who developed CRS displayed an elevated induction of CXCL10, IFN-γ, IL-6 and TNF-α upon first dosing with 0.4 mg TYRP1-TCB. C, cycle; CRS, cytokine release syndrome; CXCL10, C-X-C motif chemokine ligand 10; D, day; EOI, end of infusion; h, hours; IFN-γ, interferon-gamma; IL-6, interleukin-6; TNF-α, tumor necrosis factor-alpha.

Overall, the magnitude of cytokine induction appeared to be dose-dependent during the first dose, and levels were higher in the flat dosing cohort, compared with the single SUD and fractionated cohorts. Additional cytokines, including IL-2, IL-8, IL-10, and soluble IL-2R were also measured and showed similar dose-dependent induction (data not shown). Participants who developed CRS displayed an elevated induction of CXCL10, IFN-γ, IL-6, and TNF-α upon their first 0.4 mg dose (**Figure 2**), which supports dose-dependent cytokine induction. At Cycle 2, participants without CRS (predominantly those from the fractionated cohort) displayed slightly elevated levels of IFN-γ and IL-6 compared with baseline. Importantly, 24 h after first infusion, the median IL-6 induction for CRS-negative participants was ~5-fold compared with ~26-fold for CRS-positive participants (**Figure 2**). CRS-positive participants experienced an 18.5-fold higher median increase in CXCL10 compared with CRS-negative participants; for IFN-γ and TNF-α, the increase was 20-fold and ~2-fold higher, respectively, in favor of CRS-positive participants.

In addition to plasma cytokines, intratumoral CD8 changes were evaluated following treatment with TYRP1-TCB. Six (flat dosing, *n = 3*; single SUD, *n = 2*; fractionated dosing, *n = 1*) sets of paired biopsies (measured at baseline and at Cycle 2 Day 8) were obtained and evaluated. Despite the limited number of samples, two participants (#5 and #20) had an increase in CD8 T-cell density (>3-fold compared with baseline); samples from the other participants showed reduced or stable on-treatment CD8 densities (data not shown).

No PD changes were observed in blood when assessing various immune cell populations (immune cell populations in blood and intratumoral CD8 changes were evaluated in paired biopsies).

### 3.6 Pharmacokinetics of TYRP1-TCB and ADA response

The mean PK parameters of active (dual TYRP1- and CD3-binding competent) TYRP1-TCB following first administration were calculated (**Supplementary Table 2**). Due to the changes in exposure observed after multiple administrations of TYRP1-TCB (associated with ADA presence), PK analysis is only shown for the first dose of TYRP1-TCB, where sufficient data are available for an effective comparison.

**Figure 3:**
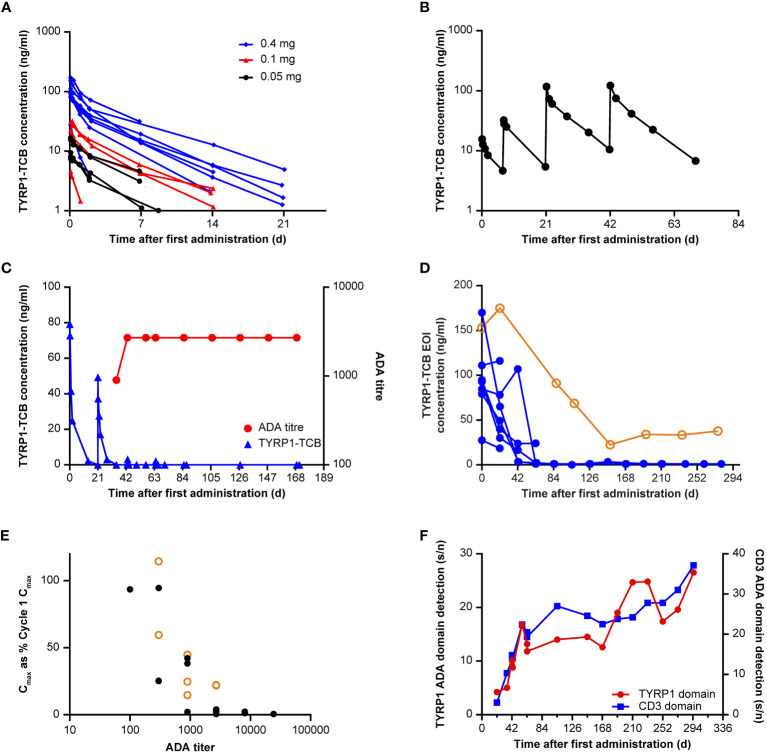
Immunogenicity and effect on exposure. **(A)** First-dose PK of TYRP1-TCB following intravenous infusion of 0.05, 0.1, and 0.4 mg TYRP1-TCB (Participants #3–20). **(B)** Example PK profile following fractionated dosing of 0.05 mg, 0.1 mg (7 days later), 0.4 mg (21 and 42 days later) TYRP1-TCB (Participant #16). **(C)** Example (Participant #7) of the effect of ADA onset (red) on TYRP1-TCB PK (blue). **(D)** Loss of exposure (assessed using EOI concentration data) across all 8 participants administered with 0.4 mg TYRP1-TCB (flat dosing) shows generality of ADA effect exemplified in element **(C)**. BLQ values set as 1 ng/mL for illustrative purposes in this figure. Participant #9 (data shown in hollow orange circles) exhibited a weaker ADA response resulting in only partial loss of active TYRP1 TCB; data from all other participants are shown using solid blue circles. **(E)** Y values: C_max_ (each administration, EOI measurement) plotted as a percentage of dose-normalized first administration C_max_ (Cycle 1 Day 1); X values: ADA titer for all ADA-positive participants (Participants #3, #4, #6–9, #13, #19). Participant #9 (data shown in hollow orange circles) exhibited a weaker ADA response resulting in only partial loss of active TYRP1 TCB; data from all other participants are shown using solid black circles. **(F)** Domain detection assay using ADA-positive sera from Participant #3 shows ADAs react to both TYRP1 and CD3 domains of the TYRP1-TCB. All other examined participants also showed ADAs targeting both the TYRP1 and CD3 domains. Y-scales: signal/noise ratios for the different assays, arbitrary units. ADA, anti-drug antibody; BLQ, below the limit of quantification; C_max_, maximum (peak) serum concentration; d, days; EOI, end of infusion; PK, pharmacokinetic; s/n, signal-to-noise ratio; TCB, T-cell engaging bispecific; TYRP1, tyrosinase-related protein 1.

C_max_ (maximum [peak] serum concentration) values ranged from 3 ng/mL at the 0.045 mg dose level, to 170 ng/mL at the 0.4 mg dose level and were attained at the end of the IV infusion. In the 8 participants who received 0.4 mg TYRP1-TCB as a flat dose, C_max_ ranged between 28 ng/mL and 170 ng/mL, with a mean (± standard deviation [SD]) of 102 ( ± 44) ng/mL. The half−life ranged between 18 and 129 h, with a mean ( ± SD) of 87 ( ± 34) h. Other parameters such as area-under-the curve, clearance, and volume of distribution are shown in **Supplementary Table 2**. First-dose PK profiles are shown in **Figure 3A**, including all participant data for the 0.05 mg, 0.1 mg, and 0.4 mg doses. Repeat administration of TYRP1-TCB resulted in consistent and dose-proportional PK profiles in the absence of ADA response, as illustrated in **Figure 3B** for 1 participant treated with the fractionated dosing schedule.

ADAs were observed in 9 of 20 participants enrolled in the study, including 6 of 8 participants who received more than four cycles of drug treatment (**Table 3**). The presence of ADAs reduced the concentrations of drug-binding competent TYRP1-TCB in serum, compared with levels observed in initial cycles. This is exemplified in **Figure 3C**, which shows PK and ADA titer data from 1 participant receiving the 0.4 mg flat dose who remained on study for multiple cycles of treatment. **Figure 3D** shows how the EOI (C_max_) concentrations changed for participants given 0.4 mg TYRP1-TCB with increasing time on study. Active drug C_max_ was reduced to ~1 ng/mL or less for all but 1 participant remaining on study by the end of Cycle 4 (84-day timepoint).

Nineteen of 20 participants had no pre-existing ADAs. A low-level pre-existing ADA response was observed in Participant #10, but there was no obvious impact, with EOI concentrations of 111 ng/mL and 116 ng/mL in Cycles 1 and 2, respectively, well within the range of EOI concentrations measured in other subjects in the same cohort. Unfortunately, Participant #10 did not continue in the study after Cycle 2 so additional assessment was not possible. ADAs were observed in 9 participants, with first detection occurring in Cycle 2–4; ADA presence was persistent in all subsequent study measurements. For Participant #8 and #9, who had low initial ADA titers (100 and 300, respectively), titer values increased to within 3-fold of the maximum titer by the start of Cycle 4. The other participants titer values remained constant ( ± 3-fold of titer value, 1 dilution step). Dose-dependence of ADA formation could not be assessed in the study as only two participants were enrolled with target doses other than 0.4 mg.

Positive results from initial ADA screening were followed up with a confirmatory test and ADA titer determination. ADA titer values can be seen in **Table 3** in the ADA-positive timepoint cells. A clear relationship between ADA titer and PK was observed (**Figure 3E**). Across all participants and all available cycles, an ADA titer of 900 or more resulted in a greater than 50% reduction in dose-normalized C_max_ (compared with Cycle 1 C_max_). Further, for all but 1 participant (see **Figure 3E**, outlier Participant #9 data marked in orange circles), a titer of 2700 or more caused more than 95% reduction in active drug dose normalized C_max_. Domain characterization assays showed that ADAs are bound to both the TYRP1-binding and CD3-binding domains (**Figure 3F**). There was noticeably lower ADA incidence in the single SUD and fractionated dosing cohorts compared with the 0.4 mg flat dosing cohort (**Table 3**). However, participants receiving fractionated dosing or SUD did not receive the 0.4 mg dose until Cycle 2, and therefore comparisons using an equal number of 0.4 mg administrations should be made using data from one cycle later for these participants. Unfortunately, most of the participants in the single SUD and fractionated dosing cohorts did not remain on study long enough for a proper comparison of the relative immunogenicity of the different dosing schedules to be made.

### 3.7 Measurement of total TYRP1-TCB concentrations

**Figure 4:**
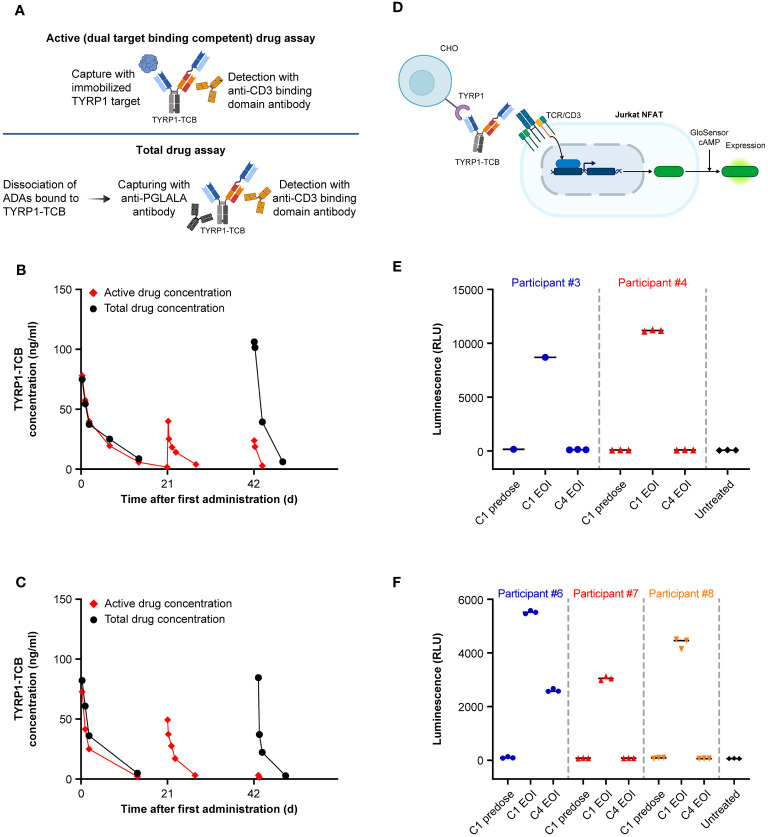
Comparison of total and active drug exposure for selected participants and *in vitro* activity of ADA-positive and ADA-negative participant sera. Comparison of total and active drug concentration in participants who developed ADAs: **(A)** Schematic of key elements of active and total drug assay methods. **(B)** Example of moderate ADA impact in Cycle 3 for Participant #6 (titer of 300 in Cycle 3 predose sample). **(C)** Example of high ADA impact in Cycle 3 for Participant #7 (titer of 2700 in Cycle 3 predose sample). **(D)** Schematic of *in vitro* activity assay. **(E)** and **(F)** Activity of participant sera before (C1 EOI) and after (C4 EOI) onset of ADA response performed in two separate experiments. Created with BioRender.com. ADA, anti-drug antibody; C, cycle; cAMP, cyclic adenosine monophosphate; CHO, Chinese hamster ovary; D, day; EOI, end of infusion; NFAT(-RE), nuclear factor of activated T cells (-reporter); RLU, relative light unit; TCR, T-cell receptor; TYRP1, tyrosinase-related protein 1.

Following on from the observed loss of active drug exposure, serum samples from ADA-positive participants were analyzed using a total drug concentration assay. This assay employs dissociation of the formed ADA-drug complexes and thus enables detection of all drug molecules (free or complexed with ADAs). Consequently, the assay allows evaluation of whether the observed loss of active exposure is due to ADA-dependent enhanced drug clearance (34) or due to drug neutralization, i.e., loss of ability to bind to its targets (**Figure 4A**). The total drug PK assay results were in concordance with the active PK (dual TYRP1- and CD3-binding) assay for Cycle 1 where the subjects were ADA-negative, but substantially different for Cycle 3 when an ADA response had developed (**Figures 4B, C**). These showed that the expected amount of total drug was present at the EOI but could not be detected using the active PK assay, which is sensitive to ADA neutralization and only detects active drug molecules.

### 3.8 *In vitro* activity of participant-derived serum samples

To decipher whether ADA-bound drug could potentially be functionally active, we carried out an *in vitro* drug activity assay using participant sera. The functional activity of sera containing TYRP1-TCB from participants who developed ADAs was tested in a Jurkat NFAT reporter cell assay with CHO-K1 TYRP1 cells (**Figure 4D**). Upon crosslinking of CD3 on Jurkat NFAT cells with TYRP1 on CHO-K1 TYRP1 cells via the TYRP1-TCB, luminescence can be measured as a marker of activation. Cycle 1 predose, Cycle 1 EOI and Cycle 4 EOI serum samples for Participants #3, #4, #6, #7, and #8 were tested in the Jurkat NFAT reporter cell assay using 70% human serum to minimize any potential dilution-mediated ADA dissociation. Cycle 1 predose serum sample (drug-negative, ADA-negative) was included as a negative control and did not show activity in the assay in any participant sera tested.

The Cycle 1 EOI sample of all tested participants (drug-positive, ADA-negative) gave a strong signal in the reporter cell assay, confirming that the functional activity of TYRP1-TCB can be measured in participant sera (**Figures 4E, F**). In contrast, Cycle 4 EOI samples (drug-positive, ADA-positive) from 4 of 5 participants did not give any measurable signal in the reporter cell assay. EOI serum samples were selected for comparison as these could be expected to have essentially the same concentration of drug present at both Cycle 1 (no ADA) and Cycle 4 (study Day 64; ADA present) and thus differ only in the presence of ADAs. Participant #6 (low titer ADA signal of 300 and diminished, but measurable, active TYRP1-TCB in serum) had a measurable, but lower, activity signal in Cycle 4 compared with Cycle 1 EOI samples. This indicated that when no active TYRP1-TCB could be measured in the serum samples (participants 3, 4, 7, and 8), functional activity was lost and ADA formation was therefore likely to impact efficacy.

## 4 Discussion

Based on encouraging preclinical data (18), we conducted this FIH phase 1 trial with a novel 2 + 1 format TCB antibody targeting the melanoma antigen TYRP1. TRAEs observed with TYRP1-TCB in participants with TYRP1-positive melanoma were consistent with its expected mechanism of action and were generally manageable across the tested dose range of 0.045 to 0.4 mg. Overall, the incidence and grade of TRAEs was similar to other CD3 bispecific molecules, such as tebentafusp and tarlatamab, where TRAEs were reported in approximately 100% of patients, including 40–60% with grade 3 or higher TRAEs (10, 35, 36). The most common TRAE was CRS, an expected on-target effect for CD3-bispecific molecules.

CRS was grade 1 or 2 and confined to the first two cycles of treatment. Fractionated dosing, i.e., starting with two lower doses before administration of the target (third) dose, prevented CRS occurrence at the administered doses in the limited number of participants tested. This is in line with the previously described tachyphylaxis effect for TCB molecules, whereby starting with one or two lower doses mediates tolerability of later higher doses (37–39). Rash as the second most common TRAE is likely an on-target (off-tumor) effect, due to targeting of TYRP1-positive melanocytes in the skin. Skin-related AEs, such as rash, have also consistently been reported as a common toxicity for tebentafusp, another TCB targeting a melanocyte-specific antigen (gp100) (10).

The increases of key peripheral cytokines, including IL-6, TNF-α, CXCL10, and IFN-γ, were temporally associated with clinical CRS symptoms in the flat dosing and single SUD cohorts. Participants in the fractionated dosing cohort, where no CRS events were reported, showed increasing levels for IFN-γ and CXCL10 similar to the flat-dosing cohort. In contrast, IL-6 and TNF-α levels seemed to be maintained over time until the target dose of 0.4 mg was achieved. This suggests that IL-6 and TNF-α are associated with the TCB-induced CRS events observed in the flat dosing and single SUD cohorts. For CD3 bispecific antibodies, it has been reported that while T cells are the main initial source of TNF-α leading to maturation of myeloid cells, monocytes, and macrophages are responsible for secreting systemic and toxic IL-6 and TNF-α involved in CRS (40). Thus, one potential explanation could be that the more frequent weekly dosing in the fractionated dosing cohort tolerized the myeloid cells, preventing the elevation of IL-6 and TNF-α. Nonetheless, the limited size of these cohorts does not support definitive conclusions regarding clinical CRS occurrence and cytokine involvement.

The prevalence of TYRP1 expression (>1%) in cutaneous melanoma observed in this study was in the same range as the prevalence in metastatic cutaneous melanoma samples from an in-house internal tissue bank (*n* = 51), analyzed with the Ventana EPR13063 investigational IHC assay (70% vs 66%). For cutaneous melanoma, Bolander et al. (41) reported a prevalence in the same range, although a different TYRP1 antibody was used in this study (41). For uveal and mucosal melanoma, other groups have reported different prevalence values than our study (~60% for uveal melanoma [14] and ~90% for sinonasal mucosal melanoma [15]); however, different TYRP1 assays were used in those studies and only primary tumor samples were analyzed. Furthermore, Jha et al. (14) used a higher cut-off (>50% staining) to define positive expression.

Development of ADAs substantially impacted the PK of active (i.e., dual target-binding competent) TYRP1-TCB. This was especially apparent in the flat dosing 0.4 mg every-3-weeks cohort where participants had the highest mean time enrolled in the clinical trial. Indeed, 6 out of 8 of these participants developed ADAs, while the other 2 remained in the study for only two cycles of treatment and so could not be assessed fully for ADA response. Participants developed ADAs towards both the TYRP1 and the CD3 domains of the molecule. This clinical study made use of an ‘active drug’ PK assay measuring the concentration of drug able to bind both TYRP1 and CD3 proteins simultaneously, and therefore capable of promoting synapse formation between TYRP1-expressing melanoma cells and CD3+ T cells. Active drug PK is the most relevant measurement for association of active drug exposure and efficacy, the potential for target-mediated drug disposition, and the impact of ADA response (42). Indeed, active drug PK assessment rapidly identified loss of active drug exposure, resulting in mechanistic follow-up, risk assessment and consideration of ADA mitigation strategies from an early stage in the dose-escalation. ADA assessment consisted of screening, confirmation, and titer determination stages. All serum samples screened positive were later confirmed as having ADAs present. The ADA titer was associated with the magnitude of ADA effect, with a very substantial impact on active drug exposure observed in all subjects with ADA titers of 900 or more. The early development of neutralizing ADAs in most participants, together with the low doses assessed in the study, might have impacted the opportunity to observe clinical activity with the molecule, even though preclinical data for TYRP1-TCB demonstrated tumor shrinkage (18). Notably, a T-cell bispecific with a similar target, tebentafusp, has recently been approved for the treatment of uveal melanoma (10).

TYRP1-TCB, like most drugs in this class, initially distributes within the volume of circulating blood and then more slowly into the body tissues. Similar C_max_ concentrations are therefore expected for any given dose at the EOI unless the drug is rapidly sequestered. An adaptive response causing substantial reduction in the target binding competent C_max_ concentrations was therefore indicative of an ADA response neutralizing the drug or enhanced drug clearance. This hypothesis was tested in the study using PK serum samples in a ‘total drug’ ligand binding assay. In this assay, proteins bound to the TYRP1-TCB, particularly the formed ADAs, are dissociated under acidic conditions and the TYRP1-TCB can be captured and measured. During Cycle 1, the total drug and active drug measurements were very similar, as expected. However, in participants who showed an ADA response and loss of active drug exposure, the total drug assay data were quite different and showed that TYRP1-TCB was indeed still circulating in the quantities expected. These data indicated that the formed ADAs bound to the drug, resulting in the observed loss of target binding competent (active) drug concentrations. Although pretreatment with obinutuzumab (an anti-CD20 antibody) to attenuate ADA responses was an option in the study protocol, this was not implemented mainly due to the concerns for B cell depletion during the emerging COVID-19 pandemic.

Insufficient PK profiles were obtained during the later cycles to determine whether or not the ADAs also increased systemic drug clearance. Previous studies on the impact of the ADA-drug immune complex size on the drug PK have shown that higher molecular weight ADA-drug complexes are very rapidly cleared from the circulation. In contrast, clearance of dimeric ADA-drug complexes was similar to the clearance of free/monomeric drug. The observation that total drug PK in the presence of ADAs was comparable to the expected PK behavior of the free/monomeric drug could indicate that dimeric complexes were preferentially formed (34, 43).

The applied Jurkat NFAT reporter cell assay to assess *in vitro* activity, confirmed that the drug present in ADA-positive samples lost its PA due to formation of ADA drug complexes. Furthermore, the observed activity loss in the *in vitro* assay confirmed that the applied dual target binding competent PK assay is indeed a true active drug assay. It enabled measurement of active drug concentrations in participant samples in this case and particularly the impact of ADA formation on active drug exposure. This example underlines the importance of clearly characterized bioanalytical methods for PK evaluation (free/active or total) and shows the benefit of a target binding competent or even active drug PK assay to sensitively determine the neutralization potential of formed ADAs. Staack et al. (44) have previously reported another example of using a cell-based activity assay to show that an appropriately developed ‘target binding competent’ PK assay reflects active drug concentrations in the participant sample (44). The sole use of a total drug PK assay might have been misleading, since it would indicate drug exposure, albeit the present drug is fully neutralized in this study.

In drug development, the incidence of ADA induction has been reduced with the introduction of chimeric monoclonal antibodies, and subsequently fully humanized or fully human monoclonal antibodies. Despite these improvements, monoclonal antibodies can still be relatively immunogenic and the reasons for this are considered to be multifactorial. Drug epitopes can be processed and presented to T cells, thereby stimulating T helper cell responses (45). Alternatively, drug epitopes can lead to T-cell independent induction of ADAs by binding to B cells, and through their differentiation, result in ADA formation (46, 47). Additionally, the participants immune status and genetic makeup may contribute to immune responses against treatment (48, 49).

Development of ADAs against protein-based immunotherapeutics, in particular bispecific cancer immunotherapy drugs, represents a common liability preventing the development of this drug class. The underlying mechanisms that lead to ADA development are not yet well understood but several efforts are ongoing to identify molecules at risk (50, 51). To this end, further research is needed to better understand the underlying biology of cognate T and B cell interactions and how this leads to ADA development and treatment resistance in people treated with biotherapeutics. A predictive tool for the development of neutralizing antibodies would be welcome.

## 5 Conclusions

Based on the totality of immunogenicity and efficacy data, the current study was terminated. Although TYRP1-TCB is currently not being developed further, the findings from this study provide valuable insights into schedule modification to avoid CRS, the assay strategy for PK analysis, and the immunogenicity of the molecule, which should inform future efforts to develop TCB antibodies for solid tumors.

## Data availability statement

The datasets presented in this article are not readily available for review. Qualified researchers may request access to individual participant level data through the clinical study data request platform (https://vivli.org/). Further details on F. Hoffmann-La Roche Ltd’s criteria for eligible studies are available here (https://vivli.org/members/ourmembers/). For further details on F. Hoffmann-La Roche Ltd’s Global Policy on the Sharing of Clinical Information and how to request access to related clinical study documents, see here (https://www.roche.com/research_and_development/who_we_are_how_we_work/clinical_trials/our_commitment_to_data_sharing.htm). Requests to access the datasets should be directed to https://vivli.org/.

## Ethics statement

The studies involving humans were approved by each center’s ethics committee or institutional review board and were conducted in conformance with the Declaration of Helsinki, International Conference on Harmonization Guidelines for Good Clinical Practice. The studies were conducted in accordance with the local legislation and institutional requirements. The participants provided their written informed consent to participate in this study.

## Author contributions

AS: Investigation, Writing – review & editing. EM: Investigation, Writing – review & editing. AI: Formal analysis, Investigation, Methodology, Writing – review & editing. JB: Formal analysis, Investigation, Methodology, Writing – review & editing. GA-Y: Investigation, Validation, Writing – review & editing. OB: Investigation, Writing – review & editing. IS: Investigation, Writing – review & editing. MS: Investigation, Writing – review & editing. VG: Investigation, Writing – review & editing. MM: Investigation, Writing – review & editing. MC: Investigation, Writing – review & editing. RD: Investigation, Writing – review & editing. CK: Investigation, Writing – review & editing. PU: Investigation, Writing – review & editing. NJ: Formal analysis, Investigation, Methodology, Writing – review & editing. FH: Investigation, Methodology, Writing – review & editing. LF: Formal analysis, Investigation, Writing – review & editing. EO-H: Formal analysis, Investigation, Methodology, Writing – review & editing. IW: Formal analysis, Investigation, Methodology, Writing – review & editing. CB: Investigation, Methodology, Writing – review & editing. RS: Investigation, Methodology, Writing – review & editing. VK: Formal analysis, Investigation, Methodology, Writing – review & editing. SF: Formal analysis, Investigation, Methodology, Writing – review & editing.
