# Supplementary material for: Phase 1, first-in-human study of TYRP1-TCB (RO7293583), a novel TYRP1-targeting CD3 T-cell engager, in metastatic melanoma: active drug monitoring to assess the impact of immune response on drug exposure
Source: Front Oncol. 2024 Mar 21;14:1346502. doi: 10.3389/fonc.2024.1346502 (PMC10991832; doi:10.3389/fonc.2024.1346502)
Supplement: Supplementary file 1 [file DataSheet_1.docx]

# Supplementary information

# 1 Supplementary methods

## **TYRP1 expression correlation analysis**

An additional prevalence analysis was performed on *n* = 48 commercially purchased cutaneous primary and metastatic melanoma samples, assessing tyrosinase-related protein 1 (TYRP1) expression via immunohistochemistry (IHC) (Ventana TYRP1 investigational IHC assay, monoclonal antibody EPR13063; Abcam, Cambridge, UK), quantitative polymerase chain reaction (qPCR) and ribonucleic acid sequencing (RNAseq). Analysis revealed that overall, TYRP1 expression was variable across samples, spanning from no to very high expression. IHC, qPCR, and RNAseq showed strong concordance, with a high positive correlation of RNAseq versus qPCR (*p* = 0.97) and a high positive correlation of RNAseq versus IHC (*p* = 0.87, for TYRP1-IHC-positive samples). Conversely, 95% of TYRP1-negative samples as assessed by IHC had no to very low TYRP1-RNA expression (as assessed by RNAseq).

**SUPPLEMENTARY TABLE 1 Overview of PK, ADA, and cytokine sample collection, and blood flow cytometry.**

| **Assessment** | | **Cycle 1–3** | **Cycle 4** | **Cycle 5 onwards** |
| --- | --- | --- | --- | --- |
| **PK** | Flat and single step-up dosing | Predose EOI EOI +6h  EOI +24h  EOI +48h  Day 8  Day 15 | Predose EOI EOI +48h Day 8 | Predose*  EOI*  EOI +48h* |
|  | Fractionated dosing | Predose  EOI  EOI +6h  EOI +24h  EOI +48h  Day 8 predose Day 8 EOI  Day 8 EOI +6h  Day 8 EOI +24h | Predose EOI EOI +48h Day 8 | Predose*  EOI*  EOI +48h* |
| **Cytokine collection** | Flat and single step-up dosing | Predose  EOI  EOI +6h  EOI +24h  EOI +48h  Day 8 | Predose EOI EOI +48h Day 8 | Predose*  EOI*  EOI +48h* |
|  | Fractionated dosing | Predose  EOI  EOI +6h  EOI +24h  EOI +48h  Day 8 predose  Day 8 EOI  Day 8 EOI +6h  Day 8 EOI +24h | Predose EOI EOI +48h Day 8 | Predose*  EOI*  EOI +48h* |
| **ADA** | All schedules | Predose  Day 15 (cycles 2 and 3) | Predose | Predose |
| **Blood  flow cytometry** | Flat and single step-up dosing | Predose  EOI +24h (cycles 1 and 2)  Day 8 | Predose  Day 8 |  |
|  | Fractionated dosing | Predose  EOI +24h (cycles 1 and 2)  Day 8  Day 8 EOI +24h (cycle 1) | Predose  Day 8 |  |

*Collected every odd-numbered cycle.

ADA, anti-drug antibody; EOI, end of infusion; h, hour; PK, pharmacokinetics.

**SUPPLEMENTARY TABLE 2 Overview of the pharmacokinetic parameters of TYRP1-TCB.**

| **Cycle 1, Day 1  Dose, mg (*n*)** | **C_max_**  **(ng/ml)** | **AUC_INF_obs_**  **(h*ng/ml)** | **HL__Lambda_z_**  **(h)** | **CL_ss_**  **(ml/h)** | **Vss__obs_**  **(l)** |
| --- | --- | --- | --- | --- | --- |
| **0.045 (1)** | 3 (ND) | 80 (ND) | 24 (ND) | 564 (ND) | 19.2 (ND) |
| **0.05 (5)** | 11 (4) | 881 (392)* | 56 (20)* | 68 (34)* | 5.1 (2.5)* |
| **0.1 (5)** | 21 (10) | 2620 (297)** | 113 (41)** | 41 (6)** | 6.4 (3.1)** |
| **0.135 (1)** | 22 (ND) | 1589 (ND) | 58 (ND) | 85 (ND) | 7.0 (ND) |
| **0.4 (8)** | 102 (44) | 8490 (4715) | 87 (34) | 128 (226) | 7.6 (4.4) |

All data are mean (SD).

* 4 of 5 participant PK profiles contributed to these values.

** 3 of 5 participant PK profiles contributed to these values.

AUC_INF_obs,_ area under the concentration-time curve from time zero to infinity; CL_ss,_ clearance at the steady-state; C_max,_ maximum concentration; h, hour; HL__Lambda_z,_ terminal half-life; ND, no data; PK, pharmacokinetic; SD, standard deviation; TCB, T-cell engaging bispecific; TYRP1, tyrosinase-related protein 1; V_ss_obs,_ volume of distribution at steady state.

**SUPPLEMENTARY TABLE 3 Prevalence of TYRP1 expression by immunohistochemistry across melanoma subtypes.**

| **Melanoma subtype, % (n/N)** | **1% cut-off** | **25% cut-off** |
| --- | --- | --- |
| **All melanoma types** | 68 (141 / 207) | 51 (105 / 207) |
| **Cutaneous** | 59 (80 / 135) | 37 (50 / 135) |
| **Uveal** | 91 (41 / 45) | 84 (38 / 45) |
| **Mucosal** | 74 (20 / 27) | 63 (17 / 27) |

Cut-offs for TYRP-1 positivity applied were either at least 1% or 25% tumor cell expression at staining intensities equal to or greater than IHC 1+.

TYRP1, tyrosinase-related protein 1

**SUPPLEMENTARY FIGURE 1** Study design.


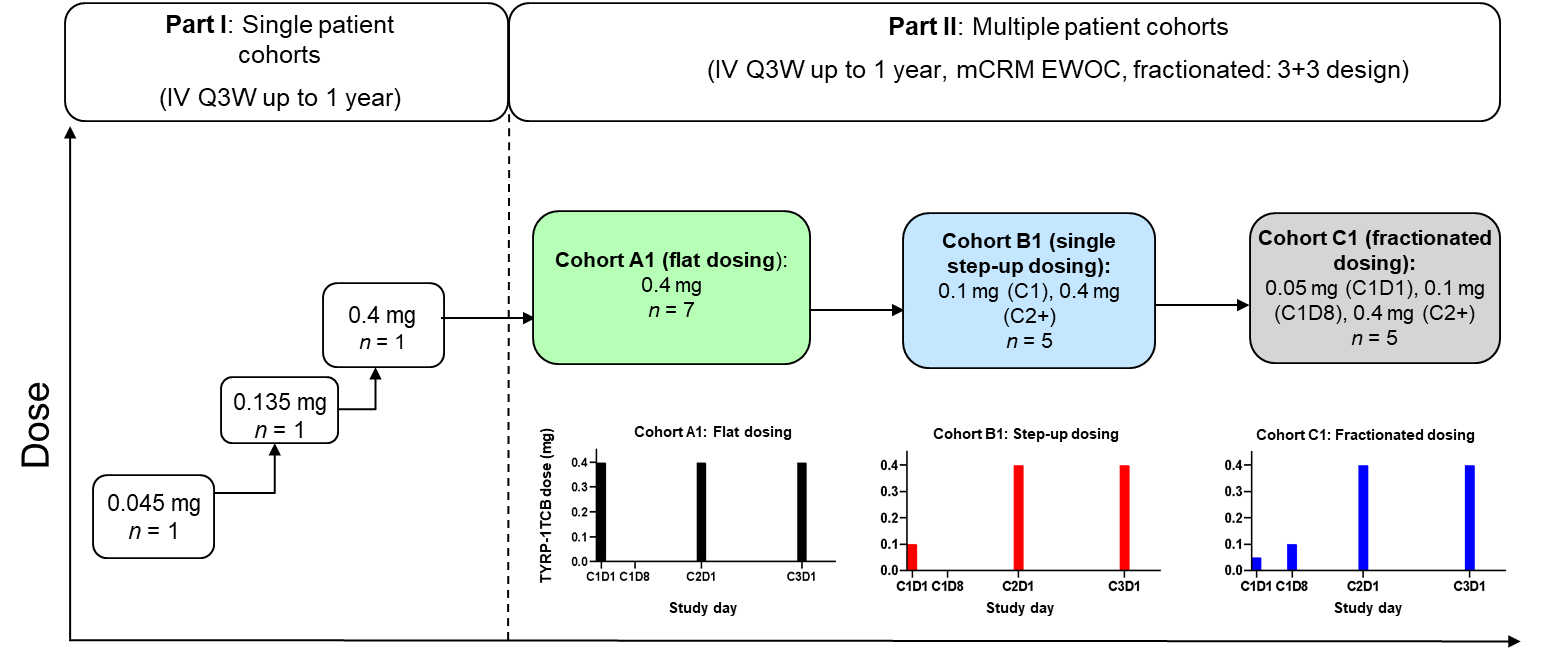


Switch from Part I to Part II occurred due to a CRS grade 2 in Participant #3. Cohorts B1 and C1 were implemented to mitigate CRS by starting with one or two lower doses. Part II was not completed, i.e., the MTD and/or OBD were not reached.

C, cycle; CRS, cytokine release syndrome; D, day; EWOC, escalation with overdose control; IV, intravenous; mCRM, modified continual assessment method; MTD, maximum tolerated dose; OBD, optimal biological dose; Q3W, every 3 weeks.

**SUPPLEMENTARY FIGURE 2** Methodology of active and total drug ELISA assays.


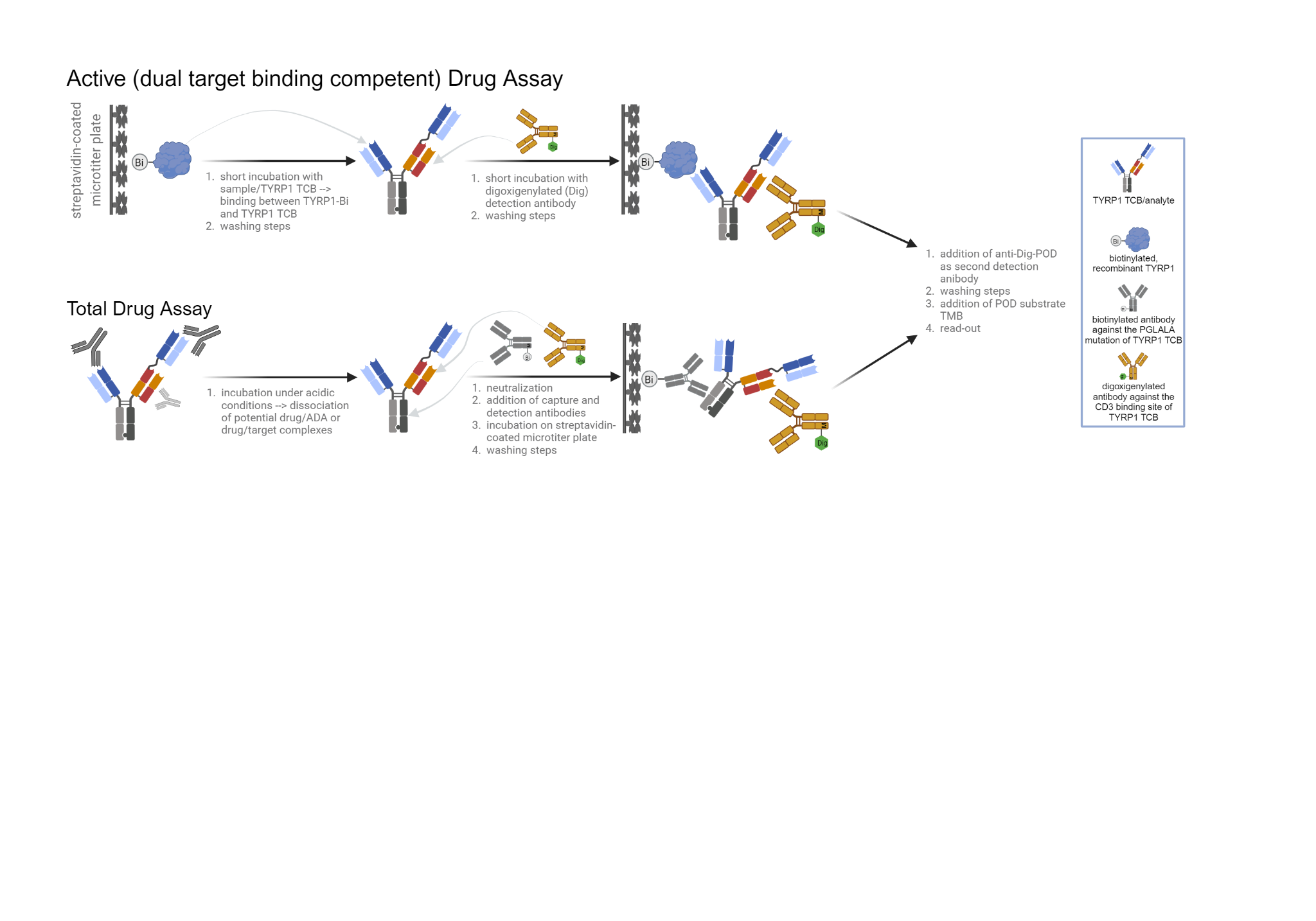


ADA, anti-drug antibody; Bi, biotinylated; Dig, digoxigenylated; ELISA, enzyme-linked immunosorbent assay; POD, perioxidase; TCB, T-cell engaging bispecific; TMB, 3,3,5,5-tetramethylbenzidin; TYRP1, tyrosinase-related protein 1; TYRP1-Bi, biotinylated recombinant human TYRP1.

**SUPPLEMENTARY FIGURE 3** Swimlane plot: duration of treatment and observed treatment response.


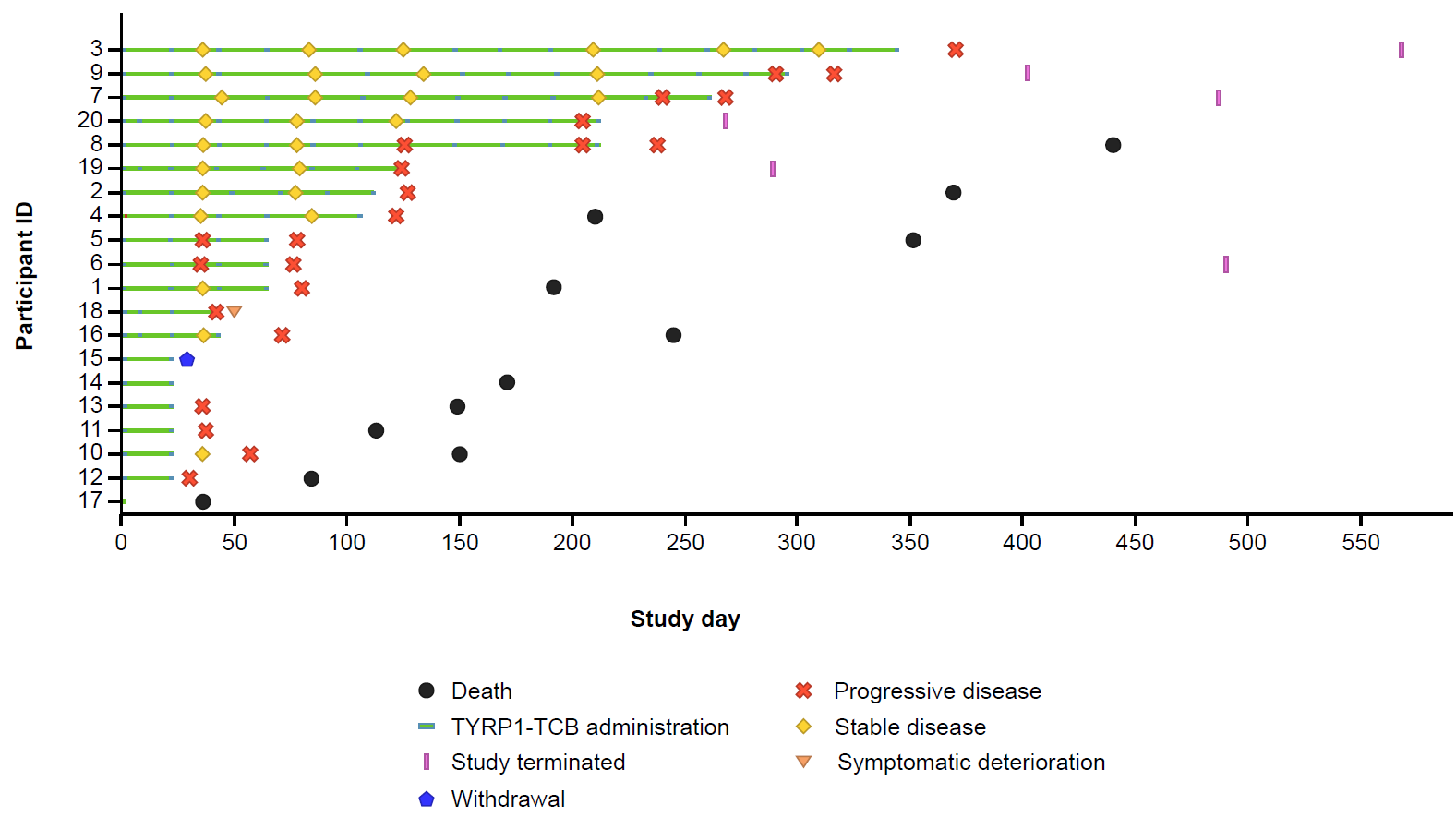


TCB, T-cell engaging bispecific; TYRP1, tyrosinase-related protein 1.
